# Experiences of Online COVID-19 Information Acquisition among Persons with Type 2 Diabetes and Varying eHealth Literacy

**DOI:** 10.3390/ijerph182413240

**Published:** 2021-12-15

**Authors:** Anna Sjöström, Senada Hajdarevic, Åsa Hörnsten, Ulrika Öberg, Ulf Isaksson

**Affiliations:** 1 Department of Nursing, Umeå University, 90187 Umeå, Sweden; senada.hajdarevic@umu.se (S.H.); asa.hornsten@umu.se (Å.H.); ulrika.oberg@umu.se (U.Ö.); ulf.isaksson@umu.se (U.I.); 2 Arctic Research Centre, Umeå University, 90187 Umeå, Sweden

**Keywords:** COVID-19, eHealth literacy, type 2 diabetes, online health information, health literacy, distributed health literacy, nursing

## Abstract

During the COVID-19 pandemic, the Internet has been a major source of information for people to keep updated with news and guidelines. However, concerns have been raised about the ‘infodemic’, which includes the overabundance of online information and the spread of misleading information. Adequate eHealth literacy skills among world citizens have therefore been emphasized as vital during the pandemic. Persons with type 2 diabetes have been at increased risk of severe outcomes of COVID-19 disease. This study aimed to explore online COVID-19 information acquisition experiences among persons with type 2 diabetes and varying eHealth literacy. Fifty-eight participants filled out the eHealth Literacy Scale (eHEALS), along with a qualitative questionnaire with free-text questions. Additionally, 10 participants were interviewed. Thematic analysis was applied to identify patterns in participants’ experiences. Two domains were identified: perceived challenges with online information about COVID-19, and coping strategies to manage challenges. The perceived challenges were: being exposed to information overload, dealing with conflicting information, and being strongly emotionally affected. The related coping strategies were: protecting oneself, trusting authorities, taking command, and using common sense. These strategies often involved triangulation of the information obtained, including participants consulting their common sense, various sources, or family and friends. This paper highlights the crucial role of authorities in delivering online information, that according to health literacy principles, is easy to access, understand, and use. Furthermore, our results reinforce the importance of diabetes nurses, as well as healthcare professionals in general, in encouraging patients to share their Internet findings, promote information from reliable sources, and deliver tailored information that suits individual needs. Because our results underline the importance of social support in eHealth literacy and the assessment of online health information, the inclusion of family and friends needs to be increasingly considered in diabetes care. Due to the small homogenous sample, the results of this study cannot be generalized. However, the reader can assess the transferability to other situations and settings based on our contextual descriptions.

## 1. Introduction

In early 2020, COVID-19, caused by the virus SARS-CoV-2, spread rapidly throughout the world, and in March, the World Health Organization (WHO) declared it a global pandemic [1]. The disease brought unprecedented challenges worldwide, including significant socio-economic burden, morbidity, and mortality [2,3]. To reduce the spread of the virus, extraordinary measures, including social and physical distancing requirements, quarantine policies and closure of schools and other gathering places, were introduced by governments worldwide [4]. In addition, in a way never previously experienced, individuals were expected to follow guidelines and take responsibility by taking control of their own health and showing solidarity with others. Reliable health information has been considered as key for people to stay updated with COVID-19 news and guidelines, and thereby act appropriately to slow the spread of the virus and protect the health of themselves and others [5].

Over recent decades, the Internet has played an increasing role in health information access, with many individuals reporting it as their primary source for health-related queries [6]. With the outbreak of COVID-19, health-related information suddenly dominated not only health websites, but also news websites and social media platforms. Both the benefits and the risks of abundant, easily accessible online COVID-19 information have been highlighted. Concerns have been raised about the so-called ‘infodemic’—the overabundance of online information and the rapid spread of misleading or fabricated news on social media and other outlets, which can create uncertainty and anxiety. Early in the pandemic, the WHO warned that the infodemic could spread faster and be as contagious as the virus itself, undermining an effective public health response [7]. With this challenge in mind, leading researchers highlighted low health literacy as an underestimated problem and emphasized the importance of public health literacy in the fight against COVID-19 [5]. Health literacy is a person’s ability to obtain, process, understand, and use health information to promote and maintain health [8]. Low health literacy is associated with poor outcomes, including poor health status, higher mortality, and high healthcare costs [9]. To correctly find, understand, critically appraise, and apply health information from the Internet or eHealth resources, a person needs adequate levels of eHealth literacy (eHL), a skill set that has been emphasized as vital during the pandemic [10].

In 2006, Norman and Skinner originally explained eHL as a complex interplay between several skills/literacies—namely traditional literacy, health literacy, media literacy, science literacy, information literacy, and computer literacy. As with health literacy, eHL is not considered a static set of skills but varies depending on situational demands and complexities [11]. Several researchers have over the years suggested new definitions of the eHL concept, arguing that the original eHL model is outdated—among other things, because of its lack of interactive web 2.0 content [12]. In an effort to understand the full range of elements relevant to the interaction between the individual and the eHealth technology systems, Norgaard and colleagues developed the seven-domain eHealth Literacy Framework (eHLF). These domains are: (1) using technology to process health information, (2) understanding of health concepts and language, (3) ability to actively engage with digital services, (4) feeling safe and in control, (5) motivated to engage with digital services, (6) access to digital services that work, and (7) digital services that suit individual needs [13].

Since the outbreak of COVID-19, people with type 2 diabetes (T2D) have been reported to be at an increased risk of hospitalization and mortality if infected with the virus [14,15]. With its high and increasing prevalence, along with its negative impact on individuals, health systems and economies, T2D is thought to be one of the most challenging health problems in the world, and the COVID-19 pandemic has been described as adding an enormous load to the already existing burden of diabetes worldwide [16,17]. Effective self-management by persons with T2D, which includes blood glucose and diet control, regular physical activity, and medication use, is considered paramount to prevent long-term health complications. Adequate health literacy AND eHL skills have been shown to be important for effective self-management of diabetes among other things, through association with levels of diabetes-related knowledge [18]. However, despite this demonstrated knowledge, research on the information-seeking behavior and information needs of persons with diabetes is limited [19,20]. Given that the Internet has been shown to be a major information source for patients with T2D, along with eHealth options (e.g., mobile applications for monitoring blood glucose concentration, providing feedback or motivating behavioural changes) being increasingly recommended for facilitating self-management of T2D, research exploring eHL and online information acquisition of this patient group is much needed [20,21].

In accordance with medical research in general, most COVID-19 studies have taken a quantitative approach, whether investigating biological and epidemiological aspects, or, as in this study, eHL and information acquisition. However, qualitative research is complementary and equally important, as it provides deeper insights into people’s needs, experiences, and perceptions [22]. A review of literature concerning experiences of online health information acquisition among persons with T2D rendered a few qualitative studies [23,24]. Understanding the information-seeking behaviour and eHL of persons with T2D can provide valuable knowledge to stakeholders and healthcare professionals for improving information and care given to this group [25]. Additionally, having knowledge about people’s experiences of online information acquisition in a pandemic context is vital for improving future communication strategies [26]. Therefore, this study aimed to explore online COVID-19 information acquisition experiences among persons with T2D and varying eHealth literacy.

## 2. Materials and Methods

### 2.1. Study Design

This study had a qualitative design with an inductive thematic approach. It is part of a larger project, iSMS, which aims to qualitatively and quantitatively evaluate a digital self-management support intervention among people with T2D in northern Sweden [27]. The present study focuses on eHL and uses qualitative data from in-depth interviews and free-text answers from questions added to the eHealth Literacy Scale (eHEALS) among participants in the control group. The use of surveys with open-ended questions is increasingly recognized in qualitative research. It is considered appropriate when conducting research with a focus on people’s experiences, where there is a need for depth, and there is diversity of information within the research topic [28]. The consolidated criteria for reporting qualitative research (COREQ) were used to report this study [29].

### 2.2. Participants and Data Collection

The inclusion criteria for the iSMS study, and thereby this study, were being aged ≥18 years, being diagnosed with T2D within the last five years, speaking Swedish, and owning a smartphone.

In April 2020, the 107 eligible persons were contacted by post and email and asked to score the eHealth Literacy Scale (eHEALS), the most widely used instrument for measuring eHL [30]. Participants were classified into low/high eHL groups based on their scores, with a cut-off for high eHL set at ≥26 [31]. In addition, questions regarding demographic information (age, gender, and level of education) and free-text questions were collected, inspired by key dimensions in the eHealth Literacy Framework by Norgaard et al. [13]. After two reminders, a total of 88 persons (82.5%) had answered the questionnaire. Fifty-eight of them (66%) answered the additional free-text items in the questionnaire. In addition to being asked to fill in the questionnaire, participants were also invited to be interviewed. Ten participants volunteered to be contacted for an in-depth interview. Following the analysis, we found that the distribution of demographics and eHL levels were similar between the participants in this qualitative study and the total sample of the larger project. The definition of ‘online COVID-19 information on the Internet’, which was communicated to all participants, included all COVID-19 information they had passively or actively come across from all online sources—for instance, news websites, social media platforms, government/authority websites, and health websites. Sample demographic characteristics of participants who filled out questionnaires and who were interviewed are separately presented in Table 1.

The interviews were conducted by the first author (AS) by telephone, in adherence to current guidelines regarding physical distancing. They took place in late May 2020, lasted between 11 and 52 (median 29) min, and were recorded and transcribed verbatim. The interviews were based on a semi-structured interview guide with questions focusing on perceptions and experiences of using the Internet for health information in general as well as their diabetes in particular, and also specifically for information about COVID-19. Free-text items in the questionnaire and the main questions of the interview guide are presented in Supplementary Materials.

### 2.3. Data Analysis

MAXQDA 2020 (2019, VERBI Software, Berlin, Germany) was used to store, explore, and organize qualitative data from the free-text survey and the transcribed interviews. During the initial analysis, no distinction was made between eHL groups. However, when attempting to discriminate between eHL levels using the mixed-methods function from MAXQDA, some distinct differences between groups could be identified. These differences are presented in the discussion as incidental findings. We have also identified the eHL group for each of the supporting quotations in the results.

The analysis was conducted using an inductive thematic approach, as described by Braun and Clarke [32]. Themes are described as ‘patterns of meaning’ that are identified across a data set. The six phases of the thematic approach described by Braun and Clarke were followed, namely: (1) data familiarization, (2) generating initial codes, (3) searching for themes, (4) reviewing themes, (5) defining and naming themes, and (6) producing the report. The initial analysis was performed separately by the first author and then discussed to achieve consensus in the whole research group. Mind maps were used to explore and display relationships between themes in different domains. Confirmability was secured using field notes, a reflexive journal, and an audit trail of initial interpretations and decisions before confirming the final themes. Data saturation was reached after eight interviews, as no new information related to the study aim emerged and data got repetitive. Two additional interviews were conducted in which data saturation was confirmed.

### 2.4. Ethical Considerations

The study was approved by an Ethical Review Board (Dno 2014/179-31) with a complementary application regarding expanded data collection. All participants were informed about the study before giving their written informed consent. Transcripts were anonymized, and the participants were ensured confidentiality and were free to withdraw at any time. All research data (e.g., audio recordings, transcripts, and text) were stored securely. All steps were managed in concordance with the General Data Protection Regulation [33].

## 3. Results

The purpose of the study was to explore online information acquisition experiences about COVID-19 among persons with T2D and varying eHL. Overall, the participants perceived that the online information flow, their information acquisition, and their emotional reactions had fluctuated and altered during the initial months of the COVID-19 pandemic.

Two domains were identified: perceived challenges with online information about COVID-19, and coping strategies to manage challenges. These domains contained three and four themes respectively, presented in Table 2.

### 3.1. Perceived Challenges with Online Information about COVID-19

Participants considered COVID-19 information in general as crucial to reduce the spread of the virus. They described advantages with the Internet as a source, including the ease of access and the possibilities of choosing among multiple online sources. However, they emphasized the challenges and problems associated with online information about COVID-19. Challenges included information overload and conflicting information, as well as being strongly emotionally affected by COVID-19 information. These challenges are described in further detail below.

#### 3.1.1. Being Exposed to Information Overload

Participants expressed that the online information flow about COVID-19 was too abundant, repetitive, and hard to counter. Being exposed to this flood of information was recurrently depicted as ‘drowning (them) in information’, inducing stress and making them feel dejected. COVID-19 was perceived to have taken over the Internet—social media as well as news media. News websites’ unilateral focus on COVID-19 was described as a source of irritation because the usual news topics were put aside.

Participants who used news notifications on their smartphones or tablets described it as the right way to keep updated in real-time, as situations and guidelines were perceived to alter quickly. However, these frequent notifications could also be described as stressful.

Identifying the essential content within the massive supply of COVID-19 information was perceived as tricky, and the large quantity of information was therefore described as confusing.

*A large amount of information is not always something good. I feel that the more information I obtain, the more perplexed I get. (Interviewee 1, low eHL).*

#### 3.1.2. Facing Conflicting Information

This theme encapsulates challenges concerning management and assessment of the content of online COVID-19 information sources. Participants described frustration related to the general COVID-19 knowledge gap, which hindered their desire to obtain specific knowledge. This especially applied to some participants’ perceptions of inadequate, inconsistent, and ambiguous online facts about T2D as a risk factor of greater COVID-19 severity. In contrast, other participants claimed that the knowledge of being in a risk group was sufficient.

Contradicting statements from lay persons and experts in news and social media were experienced as confusing and frustrating. When describing difficulties with assessing the credibility of online information, participants often worried about ‘other people’ who might have problems due to conflicting information and disinformation. News websites’ reports on the COVID-19 pandemic were depicted as hysterical and sensationalist. They were perceived to present stories either with ‘happy endings’ or the worst possible outcomes, and nothing in between. News media articles were described as presenting second-hand information, such as interpretations of facts by a journalist who could have potentially misinterpreted data or added their own touch. In contrast, broadcast press conferences held by the government or the Public Health Agency of Sweden were highlighted as the preferred sources of COVID-19 information, since they offered information directly from the primary source.

*There are exaggerated news reports. The media loves to inflate all possible news when given the opportunity. (Survey-A2, high eHL).*

#### 3.1.3. Being Strongly Emotionally Affected

The entire COVID-19 situation was described as affecting the participants’ emotional lives negatively, and they considered online news as a key trigger for emotions being pushed in different directions. The emotional response was expressed as depending on the nature of the obtained fact, as bad news (e.g., high death rates) evoked anxiety, and good news (e.g., lower death rates or scientific progress) evoked feelings of hope. News or facts that were personally relatable and could be applied to their own lives—for example, concerning diabetes or specific attributes applicable to themselves or their family members—were expressed as evoking strong emotional responses. The emotional influence of news reports was stated to lack parallels with the participants’ previous experiences. Considering the perceived influential role of online news, many participants expressed a desire for news media to offer more positive and hopeful angles on the pandemic.

Participants also described a gradual change in their emotional reactions during the initial months of the pandemic. Many participants indicated they had become jaded from being on tenterhooks and experiencing strong emotional reactions to news and facts at the beginning of the pandemic.

*You get fed up with all these death rates. It’s just… You end up getting blind to it and kind of stop caring. But it doesn’t feel right. It’s terrible to say, but it gets kind of worn out. I mean, it’s horrible with all these people dying, but you are like: ‘Ok, only 32 deaths today.’ Somehow, you get a little jaded. (Interviewee 3, high eHL).*

### 3.2. Coping Strategies to Manage Challenges

This domain encompasses the participants’ coping strategies for managing perceived challenges with online information about COVID-19, which involved self-protection and trust in authorities, as well as the necessity of taking command and using common sense. These coping strategies are described more in detail below.

#### 3.2.1. Protecting Oneself

This theme concerns strategies to protect oneself from negative emotions—including anxiety, confusion, and a feeling of ‘being fed-up’—caused by the online COVID-19 information flow. From spending a lot of time reading about COVID-19 on the Internet during the early phase of the pandemic and assessing most content as important, participants described gradually starting to sift through the flow and only read what they considered relevant—information about applicable guidelines and facts pertinent to their own lives, for example, information concerning diabetes. Some participants argued that other information could be ignored if one kept up to date with current codes of conduct. Participants also described a gradual shift of focus from nearly exclusively consuming COVID-19-related material when online, to actively choosing material unrelated to the pandemic, or even totally avoiding it. Many participants expressed reaching a point when they had more or less actively decided to limit their information acquisition about the pandemic for the sake of their mental well-being.

Participants described various strategies for limiting their consumption of online information, such as using time limits or avoiding COVID-19-related news before going to sleep. Some participants actively decided to avoid conducting COVID-19-related online searches, as it was experienced to cause or increase confusion and anxiety. In addition, searching for answers online was often described as generating even more questions because of the general knowledge gap around the pandemic.

*I have NOT done searches about corona and diabetes, and I think it is out of fear. Fear of reading something that I don’t want to know about myself. Because I also have trouble accepting that I have diabetes. It’s a defence mechanism. I put my head in the sand. That’s it. I can’t handle it. (Survey-E8K, low eHL).*

#### 3.2.2. Trusting Authorities

To deal with contradictory and doubtful information, many participants described taking a stance on trusting information originating from authoritative sources, such as the government or the Public Health Agency of Sweden—information they described as sensible, trustworthy, and safe. This information was preferably obtained through broadcast press conferences or televised news, as well as from authoritative websites and reports on news websites. Authoritative information was used as a base to simplify online information management and prevent confusion regarding what information to trust. This coping strategy implied that information contradicting that from which was obtained from authorities could be immediately rejected. Some participants considered sources other than authorities as pointless.

*Well, in the beginning, quite a few professors gave their opinion in the media and had quite a lot to say, and when reading it, you like… Well, you got a little ambivalent. You have to take a stand on whom to trust. And that doesn’t necessarily have to always be correct, but you sort of need something to hold on to, because otherwise, it gets very shaky. (Interviewee 4, low eHL).*

#### 3.2.3. Taking Command

This theme describes how participants coped by taking command of evaluating the credibility of online information about COVID-19. Participants using this strategy emphasized the value of being critical of sources and taking a sceptical approach. They described paying attention to the source of information and choosing online sources with a good reputation, while rejecting what they labeled ‘tabloid media’. Participants emphasized the importance of always being aware of possible disinformation or alternative agendas, such as sensational headlines or political statements to attract page views. Some participants described more advanced and investigative methods, such as using Google searches to find the source of obtained information or comparing and synthesizing information from different online sources and media types.

Most participants reported that they frequently discussed the veracity of Internet findings with relatives and friends. The motivation for these discussions could be to get an opinion from someone with more medical knowledge or to exchange perceptions with peers.

*Well, you read something but think, ‘Well, this cannot be true. Is it supposed to be like this?’ Then you try to juggle thoughts and discuss with someone, like, ‘What do you think?’ One gets a little unsure and might need confirmation of the feeling that the information is not credible… And then if the other one agrees, you mutually decide that it isn’t true.You kind of need support, a sounding board. (Interviewee 2, high eHL).*

#### 3.2.4. Using Common Sense

This theme illustrates participants’ trust in their inner ability to manage online information on COVID-19 by themselves, using common sense. Character traits, such as having a positive mindset or readily accepting new situations were experienced by some participants as helpful in staying balanced and calm when assessing information about COVID-19. When describing the process of critically evaluating the veracity of this information, many participants referred to using their common sense. Defining common sense was perceived as difficult, but was described by some participants as being connected with the knowledge and experiences one had acquired in life. Many participants referred to their work experience (e.g., healthcare, statistics, or research) as an asset when obtaining and evaluating information about COVID-19. Descriptions of common sense also included intuition—a gut feeling or an inner feeling of what is true or false. Participants emphasized the importance of having confidence in their competence and trusting their ability to evaluate this information to feel safe and in control of the current situation.

*I trust my common sense. I use my previous experiences, from life and from contacts I have had with healthcare. And also from my educational background, like risk analyses and things like that. I immediately think along these lines. In the light of my own life experience and knowledge acquisition, I feel somewhat in control of the situation. (Survey-A17, high eHL).*

## 4. Discussion

The findings from this study reveal challenges and strategies experienced by persons with T2D, belonging to a risk group, when actively and passively facing the supply of online information about COVID-19. With the increasingly digitalized nature of healthcare worldwide, these insights into people’s experiences can be useful for authorities and healthcare professionals to improve health communication to world citizens, in both pandemic and non-pandemic contexts.

One major challenge for our participants was the perception of being exposed to too much information—‘information overload’. Concurring with our results, research conducted before and during the COVID-19 pandemic has described perceived information overload to be associated with adverse psychological outcomes, including feelings of losing control, stress, anxiety, frustration, and anger [34,35]. The literature also describes perceived information overload as making it harder for individuals to identify relevant information [36]. According to our participants, such difficulties were reinforced by the contradictory nature of online information about the COVID-19 pandemic. Similarly, a recent qualitative article by Sykes et al. described problems with making sense of COVID-19 information because of the general knowledge gap, and the large volume and conflicting nature of the information. In agreement with our findings, Sykes et al. also described online COVID-19 information as having a negative impact on the emotions of participants [26]. Further, the COVID-19 disease itself has been shown to negatively influence the mental health of world citizens, causing anxiety, stress, and depression, and the surrounding infodemic has been described as fueling this effect [37]. On the other hand, adequate health literacy has proved to be a protective factor against negative emotional outcomes, such as depression and fear of COVID-19 during the pandemic [38].

After initial analysis looking for potential differences between eHL groups, we found that the single evident difference in the challenge domain was that the high eHL participants had to a greater extent actively searched for online information about COVID-19 in relation to diabetes. Furthermore, they expressed dissatisfaction about the lack of consistent information about the same. Thus, the high eHL participants experienced an information need that was not matched by the available online supply. This finding is noteworthy in light of previous research showing that patients with T2D in general have similarly high or even higher information needs than people with other chronic conditions [19].

Coping strategies used by the participants in our study to manage challenges included protecting oneself, trusting authorities, taking command, and using common sense. These strategies to protect the participants’ psychological well-being, including limiting time spent consuming online information and using a passive approach to the information flow, are supported by research showing that passive information acquisition from news media is beneficial for psychological well-being. In contrast, active news consumption (e.g., using news alerts or making frequent online searches) and excess time spent engaging with media and COVID-19, is associated with anxiety and stress [39,40]. Prior research has described persons with T2D as passive information receivers rather than active information seekers. In daily life, such passive information reception implies collection of information that happens to be available, for example, being informed by healthcare professionals, reading an information brochure in a waiting room, or coming across diabetes information when watching television [25,41].

Most participants in this study described that they had decided to trust authoritative information to manage contradictory or overabundant information. The benefits of this strategy as perceived by the participants are supported by research showing that media exposure in the form of speeches from experts and authorities was associated with positive psychological outcomes during the COVID-19 pandemic [39]. Medical information from governmental and medical authority sources is generally considered accurate and trustworthy, and Swedish society is often characterized by a relatively strong trust in governmental agencies [42]. However, contrary to our results, Sykes et al. described scepticism towards governmental COVID-19 information among their participants [26]. Prior studies have indicated an association between low health literacy and low trust in information from governmental and medical webpages, although analysis of our results did not reveal any such distinction between groups [43,44]. In both pandemic and non-pandemic contexts, our findings underline the importance of authorities, media stakeholders, and healthcare professionals in compensating for the effects of low health literacy in populations by communicating according to health literacy principles—that is, by developing and disseminating information that is easy to access, understand, and use [5].

Our results indicate that active approaches to online information acquisition, including online searching and online cross-checking of sources to verify the information, were more common among participants in the high eHL group. However, discussing online findings with other people was commonly reported in both eHL groups. Triangulation—that is, combining two or more sources or methods—is often seen as a method for improving the credibility and validity of scientific research findings. Consistent with our results, research has found that people also use triangulation in the management of online health information by comparing various websites or discussing online findings with healthcare professionals or peers [45]. For persons with T2D, reliance on friends and relatives in assessing complicated online health information has been shown to be important [23]. The literacy skills of the social network surrounding an individual (e.g., caregivers, family, friends, and support groups) are important as these people can act as literacy mediators, thereby enhancing health literacy levels of the individual through combined knowledge and effort, a phenomenon named ‘distributed health literacy’ [46]. Concerning individuals with T2D, distributed health literacy is said to be of particular importance as literacy mediators with higher abilities can compensate for the adverse effects of low health literacy, thus inducing positive health outcomes, such as facilitated self-care and improved glycemic control [47,48]. Therefore, based on existing research as well as our findings, the inclusion of family and friends needs to be increasingly considered in diabetes care.

As argued by Sykes et al., a note of caution in the pandemic and infodemic context is that discussing Internet findings with family and friends, rather than with healthcare professionals, might reduce the opportunity for misinformation to be corrected [26]. In order to have the chance to correct misunderstandings or misinterpretations, it is very important for healthcare professionals to actively encourage patients to share their Internet findings. Furthermore, healthcare professionals play a vital role in helping patients contextualize their Internet findings as well as promoting online information from reliable sources. By learning to detect low health literacy during patient interactions, healthcare professionals can adapt communication accordingly. Since information use and receptiveness among persons with T2D is reported to change over time depending on certain factors (i.e., the phase of the disease or different life events), it is important to support patients based on their individual needs. However, the fact that online health information seldom offers this kind of individualization demonstrates the important role of diabetes nurses and other healthcare professionals in delivering tailored information. To support patients in managing online health information, healthcare professionals need adequate eHL levels of their own. Therefore, future research should focus on eHL among both the public and healthcare staff.

Our participants emphasized that their perception and management of online COVID-19 information had changed over time, which reinforces the view of eHL as dynamic and situation-dependent, something that is particularly evident in the context of an unprecedented health crisis, such as the COVID-19 pandemic. The authors of the eHealth Literacy Framework (eHLF) have acknowledged that their framework is limited to the interaction between individuals and eHealth systems. In addition, they have suggested that aspects, such as social and cultural context, may be covered in broader understandings of health literacy or should be included in a third dimension outside the eHLF [13]. The results of this study underpin the view of eHL as a complex phenomenon that is intertwined with one’s context and social networks, and that is not restricted to personal skills and eHealth systems. To a large extent, our results are strengthened by previous research describing similar perceptions of online health information. However, there are also differences worth noting, such as cultural differences in levels of trust in government. This underlines the importance of considering cultural context in matters concerning online health information and eHL.

There are some limitations of this study that need to be considered. It is possible that persons who were particularly positively inclined towards eHealth system use and Internet information were more inclined to participate in the study. The same applies to participants who volunteered for interviews. The mean age of participants in this study was relatively high and they were mainly Swedish born with high levels of education, which could have influenced the results. Another potential limitation is that data collection through free-text questionnaires could, in comparison with interviews, render poorer narratives from participants. On the other hand, the anonymity that can be afforded when using questionnaires can encourage some participants to be more candid. Further, the use of telephone interviews, as opposed to face-to-face interviews, means that we could have potentially missed important non-verbal cues and body language. Concerning the measurement of eHL, it is important to bear in mind that the eHEALS questionnaire measures people’s perceived skills and self-confidence, rather than their actual demonstrated competencies using the Internet for health-related purposes. This means that there is a possibility that individuals may under- or overestimate their skills. Using quantitative data in qualitative studies is unorthodox, but given our sample size and the richness of our data we considered it relevant to include this aspect. Maxwell has listed several benefits of using quantitative data in qualitative research, including that it increases the internal generalization, enables identification of diversity within the studied group, and helps present evidence for the interpretation of data [49]. The relatively small sample size can be considered a limitation. However, the rich data from the free-text answers and the interviews were considered sufficient in relation to the study aim. The results cannot be generalized, but the reader can assess the transferability to other situations and settings based on our contextual descriptions.

## 5. Conclusions

The results of this study have shed light on challenges and strategies experienced by persons with T2D and varying levels of eHL, when actively and passively facing the vast supply of online information about COVID-19. With its qualitative approach, this paper offers insights that are unique regarding its focus both on patients with T2D and the pandemic context. When planning future communications strategies, in pandemic as well as non-pandemic contexts, it is useful for stakeholders to have knowledge about the challenges that online health information consumers have experienced and their strategies to manage these challenges. This paper highlights the important role of authorities as a reliable information source, in particular when information from other online sources is perceived as contradictory or overwhelmingly abundant. Therefore, authorities have a crucial role in delivering online information, that, according to health literacy principles, is easy to access, understand, and use. Furthermore, it is highly valuable for diabetes nurses as well as healthcare professionals in general, to encourage patients to share their Internet findings, promote information from reliable sources, and deliver tailored information that suits individual needs.

## Figures and Tables

**Table 1:** Demographic characteristics of participants in respective data collection groups.

	Questionnaire Sample	Interviewees
Total sample (*n*)	58	10
Sex		
Male	30 (51.7)	5 (50.0)
Female	28 (48.3)	5 (50.0)
Age, median (range)	73 (41–82)	66 (45–81)
Education		
Elementary school or less	14 (24.1)	1 (10.0)
Secondary school or vocational	22 (37.9)	2 (20.0)
University	22 (37.9)	7 (70.0)
eHEALS (mean 26.67)		
Low eHealth literacy (<26.0)	23 (39.7)	6 (60.0)
High eHealth literacy (≥26.0)	35 (60.3)	4 (40.0)

**Table 2:** Domains and themes.

Perceived Challenges with Online Information about COVID-19	Coping Strategies to Manage Challenges
Being exposed to information overload	Protecting oneself
Facing conflicting information	Trusting authorities
Being strongly emotionally affected	Taking command
	Using common sense

## Data Availability

Data and materials are available on request from the corresponding author.

## Supplementary Materials

The following is available online at https://www.mdpi.com/article/10.3390/ijerph182413240/s1, Supplementary Materials: interview guide.
