# Supplementary material for: Experiences of Online COVID-19 Information Acquisition among Persons with Type 2 Diabetes and Varying eHealth Literacy
Source: Int J Environ Res Public Health. 2021 Dec 15;18(24):13240. doi: 10.3390/ijerph182413240 (PMC8701656; doi:10.3390/ijerph182413240)
Supplement: Supplementary file 1 [file ijerph-18-13240-s001.zip › ijerph-1460909-supplementary.pdf]

Interview Guide

Describe your ordinary Internet use.

Describe situations when you have sought health-related information on the Internet.

Describe if and how you use the Internet in relation to your own health/to your diabetes.

In what way have you used the Internet for information during the COVID-19 pandemic?

How would you describe the information supply about COVID-19 on the Internet?

Tell me about your experiences of engaging with COVID-19 information on the Internet.

What benefits do you perceive with COVID-19 information on the Internet?

What problems do you perceive with COVID-19 information on the Internet?

Describe how you go about critically appraising health information in general/about COVID-19 information on the Internet.

Describe if and how you have looked for COVID-19 information in relation to type 2 diabetes.

Free-text Items of Questionnaire

Please describe your perceptions about the general information supply about COVID-19 on the Internet.

Describe your perceptions about COVID-19 related information on news websites.

If you use it, what are your perceptions about COVID-19-related content on social media?

Describe what benefits you perceive with COVID-19-related information on the Internet.

Describe what problems you perceive with COVID-19-related information on the Internet.

Describe how you are affected by COVID-19 information on the Internet.

Describe how you go about critically appraising health information/COVID-19 information on the Internet.

Describe if and how you have looked for COVID-19 information in relation to type 2 diabetes.
